# Functional Outcomes of Neglected Forearm Diaphyseal Fractures Treated Surgically: A Prospective Cohort Study

**DOI:** 10.7759/cureus.31035

**Published:** 2022-11-02

**Authors:** Gopisankar Balaji, Vijaykumar Loya, Sukruth A Patel, Govind Karunakaran

**Affiliations:** 1 Orthopaedics, Jawaharlal Institute of Postgraduate Medical Education & Research, Puducherry, IND

**Keywords:** neglected, non-union, malunion, fracture, forearm

## Abstract

Background

Unlike other injuries, adult diaphyseal forearm fractures necessitate open reduction and internal fixation. Fixation requires special attention to regaining the pre-injury length of both bones, alignment, proper apposition, and restoration of the radial bow. Although there are not many studies on surgical results for forearm fractures that have initially received indigenous therapy, there are a few studies on the functional outcomes of surgery in forearm malunion and non-union. Our study aimed to investigate the functional outcomes of neglected forearm diaphyseal fractures treated by open reduction and internal fixation.

Methodology

A total of 14 patients who presented with neglected forearm fractures between November 1, 2014, and February 29, 2016, were included. All cases underwent open reduction and internal fixation along with bone grafting. The following parameters were recorded preoperatively and at the one-year follow-up: Disabilities of the Arm, Shoulder, and Hand (DASH) score, Grace-Eversmann score, grip strength, and lateral pinch. At the end of one year, the range of motion was assessed along with the radiological assessment of the radial bow and union.

Results

This study included 14 adult patients, nine men and five women, with a mean age of 37.92 years. There was a significant improvement in the DASH score. All fractures united clinically and radiologically with the mean time to union being 13.85 weeks (12-18). There was a statistically significant change in the range of motion, grip strength, lateral pinch, and restoration of the radial bow. Of the 14 patients, seven had Good outcomes on the Grace-Eversmann score and the rest had Acceptable outcomes.

Conclusions

Surgical intervention in neglected diaphyseal forearm fractures leads to a satisfactory outcome. This is revealed by significant subjective and objective improvement both clinically and radiologically.

## Introduction

Adult diaphyseal forearm fractures are considered intra-articular fractures and should be managed surgically by obtaining anatomical reduction and stable rigid fixation. Fear of surgery, heavy plaster of Paris bandage, a prolonged period of immobilization, amputation, and sociocultural and financial reasons influence many people to approach traditional bone setters, especially in India. Though there are a few studies in the literature on functional outcomes of surgery in forearm malunion and non-union, there is no study on surgical outcomes in forearm fractures that have undergone native treatment initially [1-5].

This study was undertaken to assess the functional outcomes in surgically managed patients with forearm diaphyseal fractures, initially treated by indigenous methods and presented late to us.

## Materials and methods

This prospective study was done on patients who presented to our Orthopaedic Department with neglected forearm diaphyseal fractures from November 1, 2014, to February 29, 2016. In this study, neglected fractures included fractures that were initially treated by traditional bone setters with oil massage and bamboo splints and presented to us after a minimum of three weeks from injury. Only adult patients (>18 years old) who presented with both bone forearm fractures between three weeks and three months from injury were included in the study. Patients with suspected pathologic fractures, open fractures, associated ipsilateral fractures, or dislocations of other bones were excluded from the study.

Institute Ethics Committee approval was obtained for the study. The procedure was explained to the patients, and after approval, patients were included in the study after obtaining informed consent.

Demographic details of the patients included in the study were recorded along with the mechanism of fall, hand dominance, occupation, duration of native bandaging, and associated comorbidities. Preoperative Disabilities of the Arm, Shoulder, and Hand (DASH) score was measured along with lateral pinch and grip strength. The elbow, forearm, and wrist range of motion were measured. Grip strength was measured with a Jamar dynamometer, with the patient in a seated position and elbow by the side of the body. The contralateral extremity was taken as control. Measurements were first made in the control extremity, followed by the affected extremity, and a mean of three values was taken. Similarly, a pinchometer was used to measure lateral pinch by taking a mean of three measurements in both the control and affected extremity.

Radiologic parameters of the radial bow and ulnar variance were measured using digital radiographs on Picture Archiving and Communication System (PACS) (GE Healthcare, Connecticut, USA, Version 4.0) as outlined by Schemitsch et al. [1]. The ulnar variance was measured using the method of perpendiculars, as outlined by Kristensen et al. [2].

Most cases were operated under general anesthesia or regional block. After induction of anesthesia, patients were positioned supine with an arm-board, and a tourniquet was applied to the upper arm. In 13 of 14 patients, the radius was approached through the volar Henry approach, and in one case, the radius was approached through Thompson’s approach. The ulna was approached through a subcutaneous approach. The fractures were stabilized with either a 3.5 mm dynamic compression plate (DCP) or limited contact DCP. The ulnar variance was checked intraoperatively using an image intensifier to review whether adequate length was regained. In all cases, contralateral iliac crest bone grafting was done. The skin and subcutaneous tissues were closed with a suction drain. An above-elbow splint in the mid-prone position was applied. The postoperative protocol included antibiotics till the drain was removed, adequate analgesia, and limb elevation.

A wound check was done on days four, eight, and 14. Sutures/staples were removed on day 14. The splint was removed at two weeks, and patients were started on active and assisted active physiotherapy, which included elbow flexion/extension, pronation/supination, and wrist range of motion.

Patients were then followed up at regular intervals for one year. The time to union was noted. Union was defined as the presence of a bridging callus and three cortices union [6].

At the end of one year, the range of motion was documented. Postoperative DASH score along with grip strength and lateral pinch were noted. The Grace-Eversmann score was measured. The time to return to activities of daily living and return to occupation was noted. The radial bow and ulnar variance were measured using plain radiographs.

As the data were collected on preoperative and postoperative measures, Wilcoxon signed-rank test was used for the DASH Score, Grace Eversmann score, lateral pinch, and grip strength, which were quantitative in nature. To analyze the data obtained from the radial bow on X-ray, the ulnar variance on X-ray preoperatively and after 12 months, the paired samples t-test was used. Further, descriptive measures such as mean and standard deviation were presented for all variables by means of graphical visualization. The entire analysis was performed at a 0.05 level of significance.

## Results

This study included 14 patients, five female and nine male, with a mean age of 37.92 years (22-71 years). The demographic details, mechanism of injury, occupation, presenting complaints, native bandage settings, and associated comorbidities are shown in Table 1.

**Table 1:** Demographic details of patients. ADL: activities of daily living; T2DM: type 2 diabetes mellitus; CKD: chronic kidney disease; 2w: two wheeler

Age	Sex	Occupation	Duration of native bandage (in days)	Mechanism of fall	Side affected/dominant side (Right (R)/ Left (L))	Episodes of native bandage	Presenting complaints	Comorbidity
46	M	Shop keeper	68	Fall from 2w	R/R	6	Difficulty in ADLs	-
24	F	Homemaker	76	Fall at home	R/R	7	Deformity	-
26	M	Chef	54	Fall from 2w	L/R	3	Difficulty in ADLs	-
28	F	Homemaker	80	Fall at home	R/R	5	Pain	-
35	M	Crane operator	87	Fall from 2w	R/R	9	Deformity	T2DM
36	M	Driver	42	Fall from 2w	L/R	3	Difficulty in ADLs	-
46	M	Woodcutter	64	Workplace injury	R/R	5	Difficulty in ADLs	-
32	M	Shop keeper	62	Fall from 2w	L/R	2	Difficulty in ADLs	-
71	M	Farmer	51	Fall in farm	L/R	3	Pain	CKD
35	F	Homemaker	35	Fall from height	L/R	3	Pain	Reactive airway disease
24	M	Police officer	42	Fall from 2 w	L/R	6	Difficulty in ADLs	-
62	F	Homemaker	62	Fall at home	L/R	4	Difficulty in ADLs	CKD
44	M	Laborer	58	Fall from 2w	R/R	2	Difficulty in ADLs	-
22	F	Student	36	Fall from 2w	L/R	7	Deformity	-

The mean duration after which the patient presented to our hospital was 58.35 days (35-87 days). Most patients presented with deformity and restriction of forearm rotations. This study had four cases of malunion and 10 cases of non-union. There were no blebs or blisters except for one patient who had multiple scars due to manipulation and splinting by native bandaging, with elbow and finger stiffness. One middle-aged female had shiny skin with elbow stiffness, and another had shoulder and finger stiffness. One patient had posterior interosseous nerve palsy at presentation, which recovered on follow-up.

Radiologically, there was only one case of segmental fracture (radius), and the rest of the fractures were located in the middle third or the middle-distal third junction.

The mean intraoperative time was 160 minutes (135-210 minutes) as these were old fractures and the fracture ends had to be freshened or shortened, and the reduction was difficult.

The preoperative mean DASH score was 64.25 (47.5-85). The mean postoperative DASH score at the one-year follow-up was 33.53 (23-40.68). There was a significant improvement in the DASH score with a p-value of <0.001.

At the final follow-up, seven patients had Good outcomes on the Grace-Eversmann score with union and 80% forearm rotation; seven patients had Acceptable outcomes on the Grace-Eversmann score with union and 60-80% forearm rotation.

**Figure 1:**
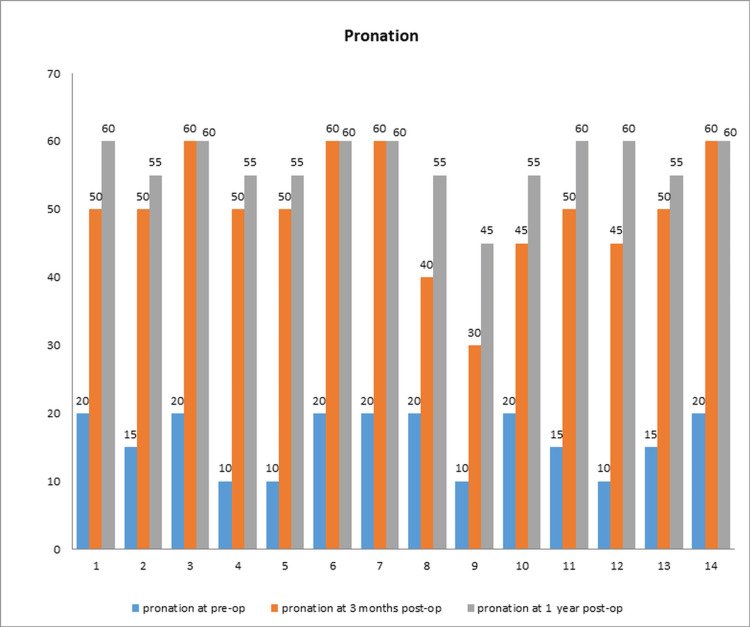
The range of pronation preoperatively and postoperatively in all patients.

**Figure 2:**
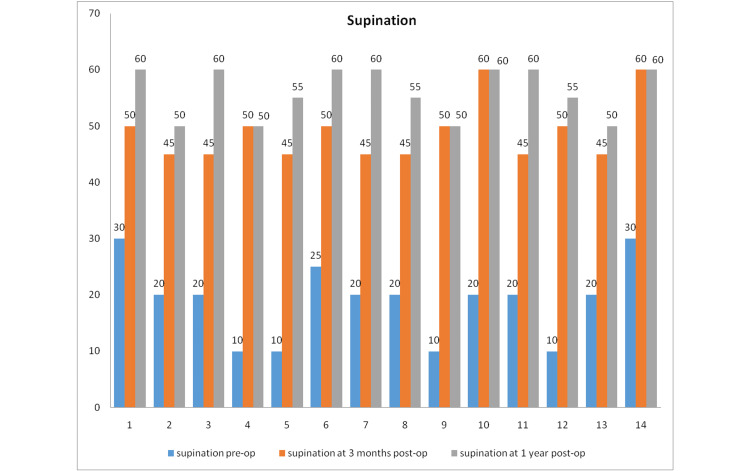
The range of supination preoperatively and postoperatively in all patients.

The mean preoperative pronation was about 16.07 degrees, and the mean supination was 18.92 degrees. The mean postoperative range of motion at six weeks was pronation at 50 degrees and supination at 48.92 degrees. At the final follow-up, pronation improved to 56.78 degrees and supination to 56.02 degrees (Figures 1, 2).

Thus, the surgical intervention resulted in statistically significant (p < 0.001) improvement in pronation and supination as measured by the Freidman test.

There was a mean 24.25% loss of pronation compared to normal (20% in seven cases, 26.6% in six cases, 40% in one case) at the one-year follow-up. The mean loss in supination was 29.01% (25% loss in eight cases, 31.25% in three cases, 37.5 in three cases).

**Figure 3:**
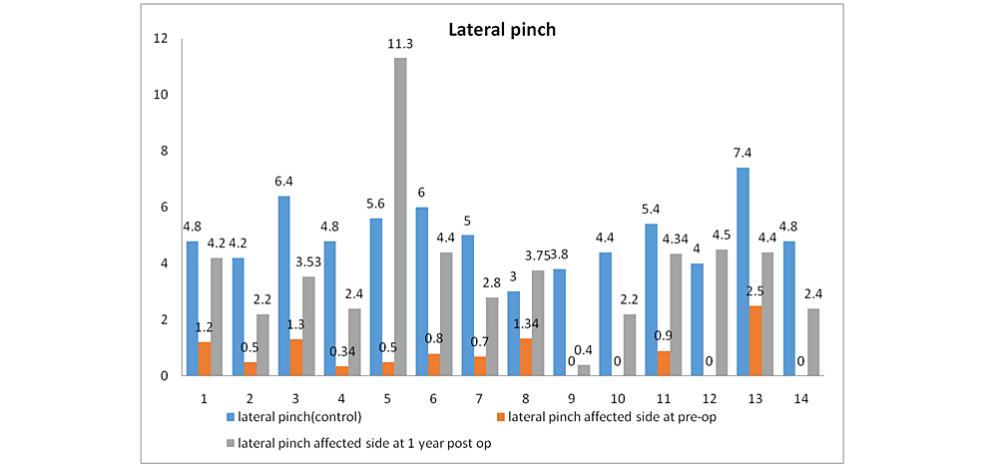
Pinch strength preoperatively and postoperatively in all patients.

The mean value for the lateral pinch of the control arm was 4.97 kg, while in the affected extremity, it was 0.72 kg preoperatively and 3.77 kg postoperatively. The Wilcoxon signed-rank test showed improvement in the lateral pinch, both preoperatively and postoperatively, with a p-value of <0.001 (Figure 3).

**Figure 4:**
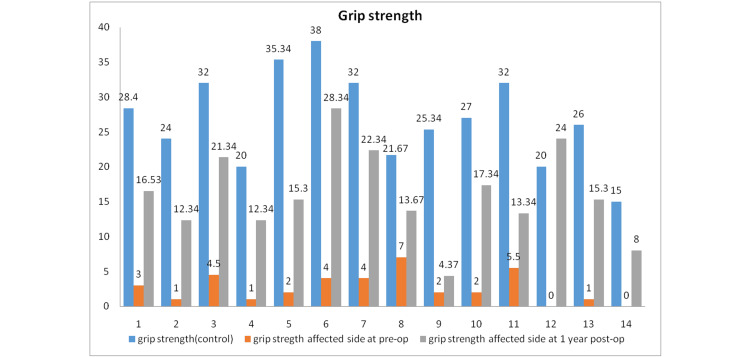
Grip strength preoperatively and during postoperative follow-up.

Grip strength was 26.9 kg in the control extremity, and in the affected extremity, it was 2.64 kg preoperatively and 16.04 kg postoperatively. Improvement in grip strength was found to be significant by Wilcoxon signed-rank tests (p < 0.001) (Figure 4).

**Figure 5:**
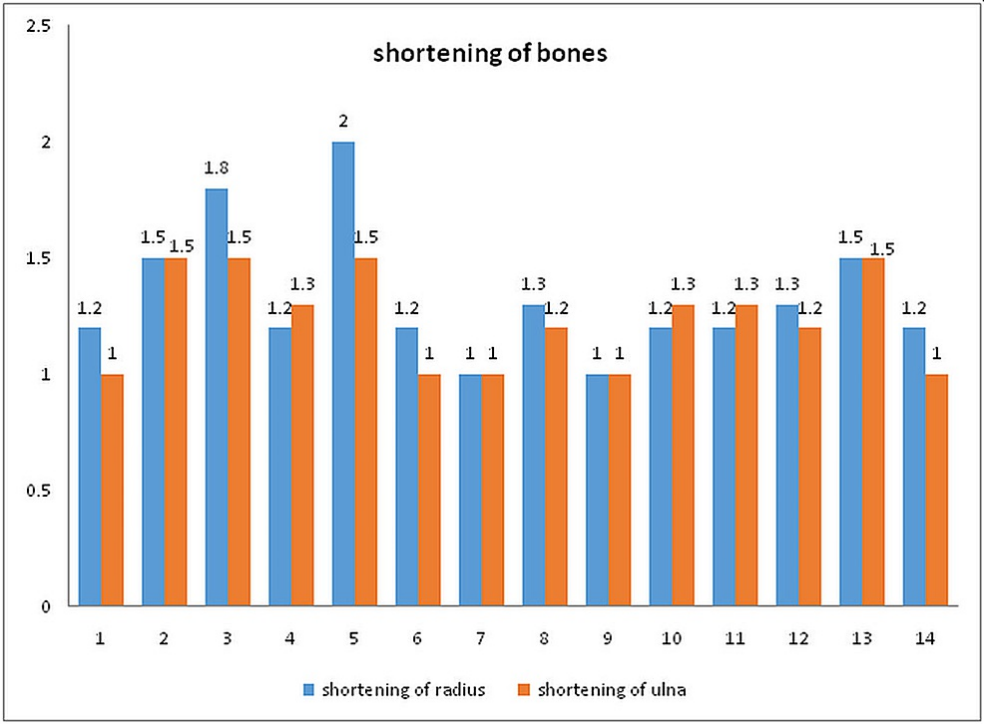
The amount of bone shortening postoperatively in all patients.

The time to union in the present study was 13.85 weeks on average (12-18 weeks). The case with segmental fracture united at 18 weeks postoperatively. The mean radial shortening was 1.33 cm (1-2 cm), and the mean ulnar shortening was 1.24 cm (1-1.5 cm) (Figure 5).

**Figure 6:**
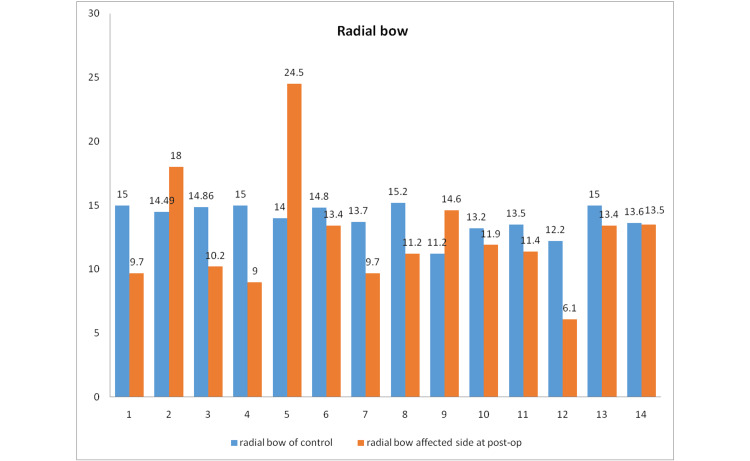
The radial bow measured radiologically preoperatively and postoperatively in all patients.

The radial bow of the control extremity was 13.92 ± 0.32 mm and the affected limb (post-intervention) was 12.61 ± 1.19 mm. Thus, the radial bow was restored postoperatively to a significant level compared to the contralateral extremity following the surgical intervention (Figure 6).

Postoperatively, the negative ulnar variance was restored in all patients except one who had a positive ulnar variance of about 10.3 mm.

Pearson correlation between radial bow and pronation was 0.14, implying that if the radial bow increases, pronation should increase; however, the correlation strength was weak.

Similarly, supination and radial bow were correlated; the Pearson coefficient of determination was 0.06 and the p-value was >0.001, signifying a poor correlation between supination and radial bow. The mean time to return to activities of daily living was 8.3 weeks (6.4-10.2 weeks), and the mean time to return to pre-injury work was 14.9 weeks (12.3-18.4 weeks).

Three patients presented initially with complex regional pain syndrome, one with scars, finger, and elbow stiffness; the second patient had shiny stretched skin with elbow stiffness; and another elderly man had shoulder and finger stiffness. The patient with segmental fracture had posterior interosseous nerve palsy, which subsequently improved postoperatively. Intraoperatively, one patient had a vertical radial artery tear, which was repaired, and one had a drill bit broken while fixing the radius plate, which was removed. Another middle-aged female had severe pain on postoperative day one after the block effect was weaned, and there was mild swelling of fingers with painful passive stretch. The slab was removed immediately and reapplied loosely, after which her pain improved and she was comfortable. One patient developed serosanguinous discharge on the first wound check, with no other evidence of infection, which subsequently subsided by day eight with regular dressings. Culture sensitivity was sterile (Table 2).

**Table 2:** Complications.

Preoperative complications	
Complex regional pain syndrome type 1	3
Posterior interosseous nerve palsy	1
Intraoperative complications	
Vertical radial artery tear	1
Drill bit breakage	1
Postoperative complications	
Development of blebs	1
Serosanguinous discharge	1

## Discussion

Forearm diaphyseal fractures in adults remodel poorly, leading to residual angulation and malunion. This results in the restriction of forearm rotations, thereby affecting activities of daily living. Jayakumar and Jupiter [3] showed a 10% decrease in forearm rotation and a 15-25% decrease in grip strength following malunion. Hence, anatomical reduction and restoration of the radial bow play an important role in regaining functional outcomes.

Although outcomes of forearm malunion/non-union have been reported in the literature, there are no studies on outcomes following surgical management in patients who underwent native bandaging initially and presented late to the hospital.

Patients in this part of the country, due to social, cultural, and economic reasons, approach traditional bone setters (or so-called “osteopaths” or puttur kata) for multiple ailments, including musculoskeletal injuries, leading to temporary complications such as skin blebs, pigmentation, superficial infections, stiffness or delayed union, and permanent complications such as malunion, non-union, or even gangrene and subsequent amputation.

Most patients were daily laborers with low educational and socioeconomic backgrounds. They traveled a long distance to reach healthcare facilities. Hence, they opted for low-cost treatment with a traditional bone setter nearby. They were unaware of the complications.

All patients presented to our hospital after undergoing native bandaging initially for a mean period of 58.35 days (35-87 days), with a mean of 4.64 settings of bandage (two to nine settings). Usually, one setting lasts around 7-15 days, depending on the bone setter. The most common presenting complaint was difficulty in carrying out activities of daily living. On presentation, one patient had multiple scars due to splinting with finger and elbow stiffness, another middle-aged female had elbow stiffness with shiny skin, and an elderly male patient had shoulder and finger stiffness. Four of the patients showed malunion, and the rest were non-union.

Few studies have analyzed patients presenting to a healthcare facility after initially undergoing treatment with the traditional bone setter. The details of these study results and complications [7-10] are listed in Table 3.

**Table 3:** A comparsion of various studies on traditional bone setters.

	Callistus et al.[7] (n = 230)	Memon et al. [8] (n = 58)	Dada et al. [9] (n = 121)	Panda and Rout [10] (n = 52)	Current study (n = 14)
Male:Female	156:74	34:14	69:52	82:65	9:5
Mean age	32.3 ± 17 (7 months to 78 years)	1–60 years	29.49 (6 weeks to 72 years)	48.6 ± 29.2 years	37.92 (22–71)
Predominant age group (years )	21–30 (23%)	1–10 (16)		21–40 (43)	20–30 (5)
Mean duration of bandage		9 weeks (3 days to 9 months)			58.35 days (3 weeks to 3 months)
Closed:Open fracture	163:67	38:07			All closed
Most common fracture	Shaft femur (24.3%)	Supracondylar humerus (24.3%)	Humerus (29%)	Forearm (17%)	Forearm (100%)
Forearm fracture	11.7%	17.2%	14.8%	17%	100%
Ankylosis	18 (7.8%)		2 (1.2%)		-
Avascular Necrosis	13 (5.7%)		9 (5.4%)		-
Contracture	11 (4.8%)	2 (3.44%)	1 (0.6%)		-
Gangrene	6 (2.6%)	4 (6.89%)	1 (0.6%)		-
Infections	39 (17%)	3 (5.17%)	8 (4.8%)		-
Complex regional pain syndrome					3 (21.42)
Malunion	72 (31.3%)	15 (25.86%)	27 (16.1)	3 (5.7%)	4 (28.57)
Non-union	48 (20.9%)	7 (12.06%)	27 (16.1)	1 (1.9%)	10 (71.4)
Paralysis	12(5.2%)				-
Compartment syndrome		6 (10.34%)	1 (0.6%)		-
Cellulitis		4 (6.89%)		5 (9.6%)	-
Heterotopic calcification			5 (3%)		-
Stiffness			18 (10.8)	3 (5.7%)	3 (21.42)

**Figure 7:**
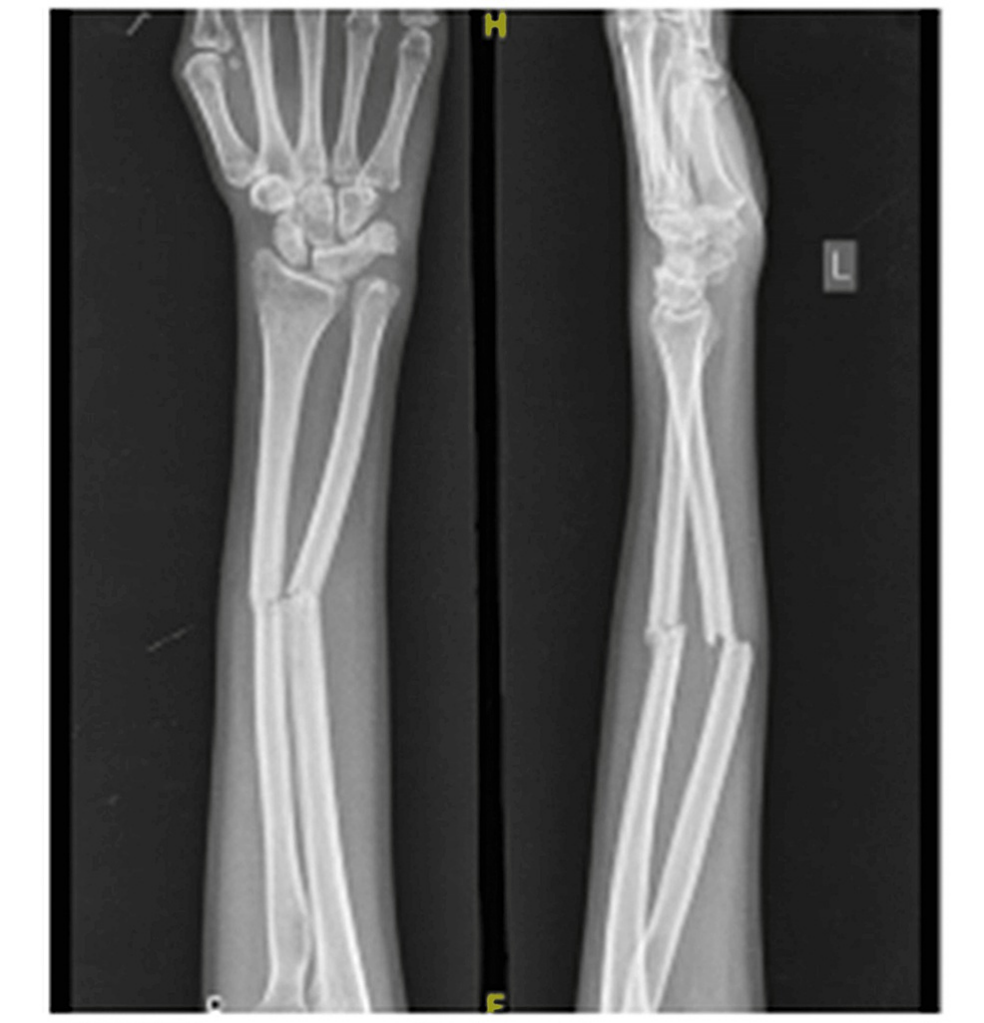
The preoperative forearm X-ray with ununited fracture of both forearm bones.

**Figure 8:**
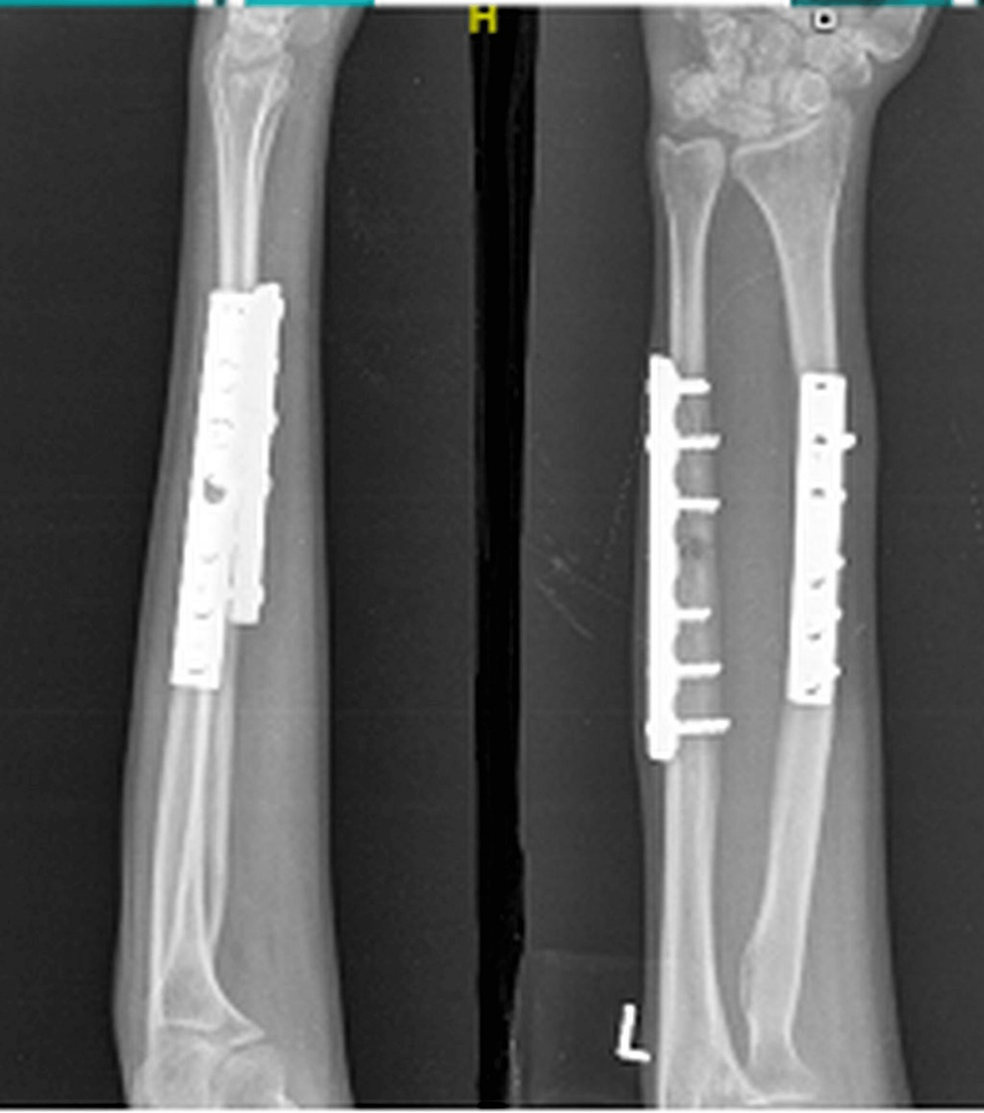
Postoperative follow-up X-ray of the patient.

**Figure 9:**
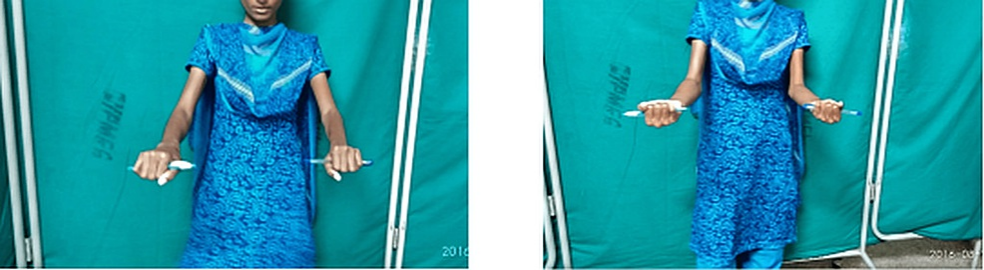
Clinical photograph of the patient demonstrating pronation and supination at follow-up.

In this study, the mean pronation was preoperatively 18 degrees which improved to 56.78 degrees one year postoperatively. Supination improved from 18.92 to 56.07 degrees in one year (Figures 7-9).

There was a 24.25% (average) loss of pronation and a 29.01% loss of supination postoperatively in the present study. Similarly, there was an improvement in objective parameters such as the lateral pinch from 0.72 kg to 3.77 kg. Grip strength improved from 2.64 kg to 26.9 kg. There was a subjective improvement in the DASH score from 64.52 to 33.53 (mean), which was statistically significant. The Grace-Eversmann score was Good in seven patients and Acceptable in seven patients.

Reis et al. studied 31 cases of non-union of the forearm that were treated by compression plating and autologous bone grafting and followed up for a minimum of two years. Fractures united in a mean time of 3.5 months, with a union rate of 96.7%. Overall, 29/31 patients had good results based on Tscherne criteria, and 26/31 resumed their work [4]. Saka et al. studied eight patients with forearm non-union with a mean age of 37 years (19-55 years). They were operated on 18 months (9-42 months) after the index procedure and followed for 32 months (18-52 months). The defect was 0.5-3 cm, and Nicoll’s technique was used for obtaining tricortico-cancellous iliac crest bone grafting. All non-union cases were treated by interlocking intramedullary forearm nails. All cases united with a preserved radial bow with a mean Visual Analog Scale score of 1 (0-3) and DASH of 10.7. The Grace-Eversmann Score was Excellent in five and Good in three patients [5].

In our study, the radial bow of control (uninjured extremity) was 13.92 ± 0.3169 mm, and that of the injured extremity at the one-year follow-up was 12.61 ± 1.192 mm, which was statistically significant. Schemitsch et al., in their landmark study, showed that for an excellent or good functional arc of rotation of the forearm (≥80%), the value of the radial bow tended to be similar to the uninjured extremity. Patients who had a more than 80% forearm rotation compared to that of the uninjured extremity had a radial bow that was 1.5 ± 0.2 mm more or less than that of normal, and those with less than 80% rotation had a radial bow of 2.8 ± 0.7mm more or less of the normal radial bow [1].

Goldfarb et al., who retrospectively studied objective and radiological parameters in 23 patients with forearm fractures treated with plating, found that the maximal radial bow was 16 ± 3 mm (10-20). They found that patients with a loss of arc of rotation of less than 10% had a mean difference of 1.9 mm (12%) from the normal maximal radial bow. Similarly, patients with a loss of rotation of 10-20% and >20% had a mean difference of 2.0 mm (13%) and 2.3 mm (15%) from the normal maximal radial bow, respectively [11].

One patient in our study had a tear in the radial artery intraoperatively while reducing the radius fracture, which was repaired. Another patient developed blebs postoperatively. One patient developed serous discharge which subsided. In the study by Reis et al., two patients developed postoperative infections, and one had to be treated with debridement and antibiotics for two weeks. The other required revision procedures, but that did not heal. No failure of fixation was seen in their study [9]. In the study by Saka et al., there were no intra or postoperative complications, no early or late infections, and no implant loosening or breakage [10]. There were no intra or postoperative complications and all cases united in a study by Faldini et al. [12]. In the study by Nagy et al., there was an alteration in rotation. However, there were no infections, delayed union, synostosis or compartment syndrome, distal radioulnar joint instability, or ulnar impaction syndrome [13].

One limitation of our study was the small sample size though each case worked as its own matched control for outcomes, minimizing individual differences if there were two different groups. This is because it was a prospective study and we wanted a single homogenous group that had initial treatment with traditional bone setters and presented to our hospital between three weeks and three months post-injury.

## Conclusions

Surgical intervention in neglected diaphyseal forearm fractures leads to a satisfactory outcome. This is shown by significant subjective improvement in the DASH and Grace-Eversmann scores. It is also proven objectively by significant improvement in range of motion, increase in the lateral pinch, and grip strength. Restoration of the radial bow near equivalent to the uninjured extremity leads to a better range of motion. Because the study’s sample size was small, further studies may be needed with similar patient profiles to corroborate the findings and identify which radiological patterns are less likely to improve with a traditional bone setter.
